# Supplementary material for: Small-molecule suppression of calpastatin degradation reduces neuropathology in models of Huntington’s disease
Source: Nat Commun. 2021 Sep 6;12:5305. doi: 10.1038/s41467-021-25651-y (PMC8421361; doi:10.1038/s41467-021-25651-y)
Supplement: Supplementary file 1 — Supplementary information [file 41467_2021_25651_MOESM1_ESM.pdf]

Supplementary information

Small-molecule suppression of calpastatin degradation reduces neuropathology in models of Huntington's disease

Di Hu[1], Xiaoyan Sun[1], Anniefer Magpusao[2], Yuriy Fedorov[2], Matthew Thompson[2], Benlian Wang[3], Kathleen Lundberg[3], Drew Adams[2,*] and Xin Qi[1,*]

[1]Department of Physiology & Biophysics, [2] Department of Genetics, [3]Proteomics Center, Case Western Reserve University School of Medicine, Cleveland, OH 44106, USA

***Corresponding author:**
Xin Qi, PhD, Department of Physiology and Biophysics, Case Western Reserve University School of Medicine, 10900 Euclid Ave, E516, Cleveland, Ohio, 44106-4970, USA. Tel: 216-368-4459; Fax: 216-368-5586; E-mail: xxq38@case.edu
Drew Adams, PhD, Department of Genetics, Case Western Reserve University School of Medicine, 10900 Euclid Ave, Cleveland, Ohio, 44106-4970, USA; Email: dja59@case.edu

Supplementary Fig. 1

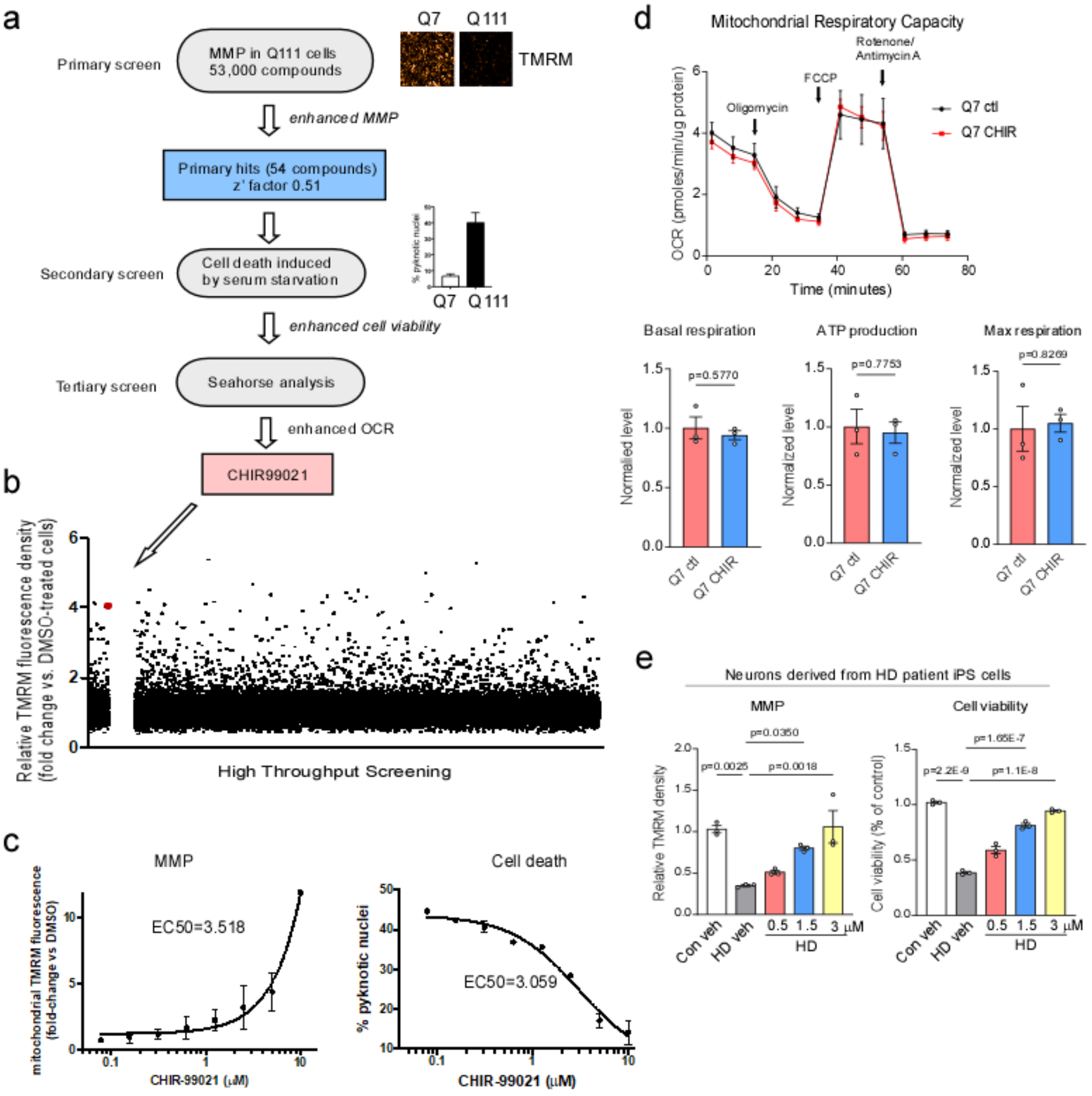

Supplementary Figure. 1. High throughput screening (HTS) identifies CHIR99021 as an enhancer of mitochondrial function in HD cells: (**a**) HTS workflow. Representative images: HdhQ7 and HdhQ111 cells stained with tetramethylrhodamine (TMRM) as a marker of mitochondrial membrane potential (MMP). Cell death was assessed by the percentage of pyknotic nuclei scored by PerkinElmer Harmony software for the Operetta high-content imaging system. OCR: oxygen consumption rate. (**b**) HTS results. Left: 3,000 bioactives; right, 50,000 diverse molecules; red dot, CHIR99021. (**c**) Left: a dose response for CHIR99021 in MMP regulation as measured by TMRM staining (n = 3); right: pyknotic cell death quantitation following treatment with indicated CHIR99021 concentrations (n = 3). (**d**) Mitochondrial respiration in HdhQ7 cells treated with CHIR99021 or DMSO (vehicle; veh) was measured using a Seahorse XFP analyzer with a Mito stress kit (n = 3). Basal respiration rate, maximal respiration rate, and ATP production are shown. (**e**) Mixed striatal neurons were differentiated from induced pluripotent stem (iPS) cells from patients with HD, (ND41656, 57 CAG) and control subjects (nHDF). Twenty days after neuronal differentiation, cells were treated with DMSO (vehicle; veh) or CHIR99021 at indicated doses. MMP was assessed as previously described (n = 3). Neuronal viability was measured by MTT assay after the withdrawal of brain derived neurotrophic factor (BDNF) for 12 h (n = 3). Data were expressed as the mean ± SEM from three independent experiments and compared using one-way ANOVA with Tukey's *post-hoc* test in e, and the unpaired student *t*-test (two-tailed) in d. Exact p values are shown in the figures.

Supplementary Fig. 2

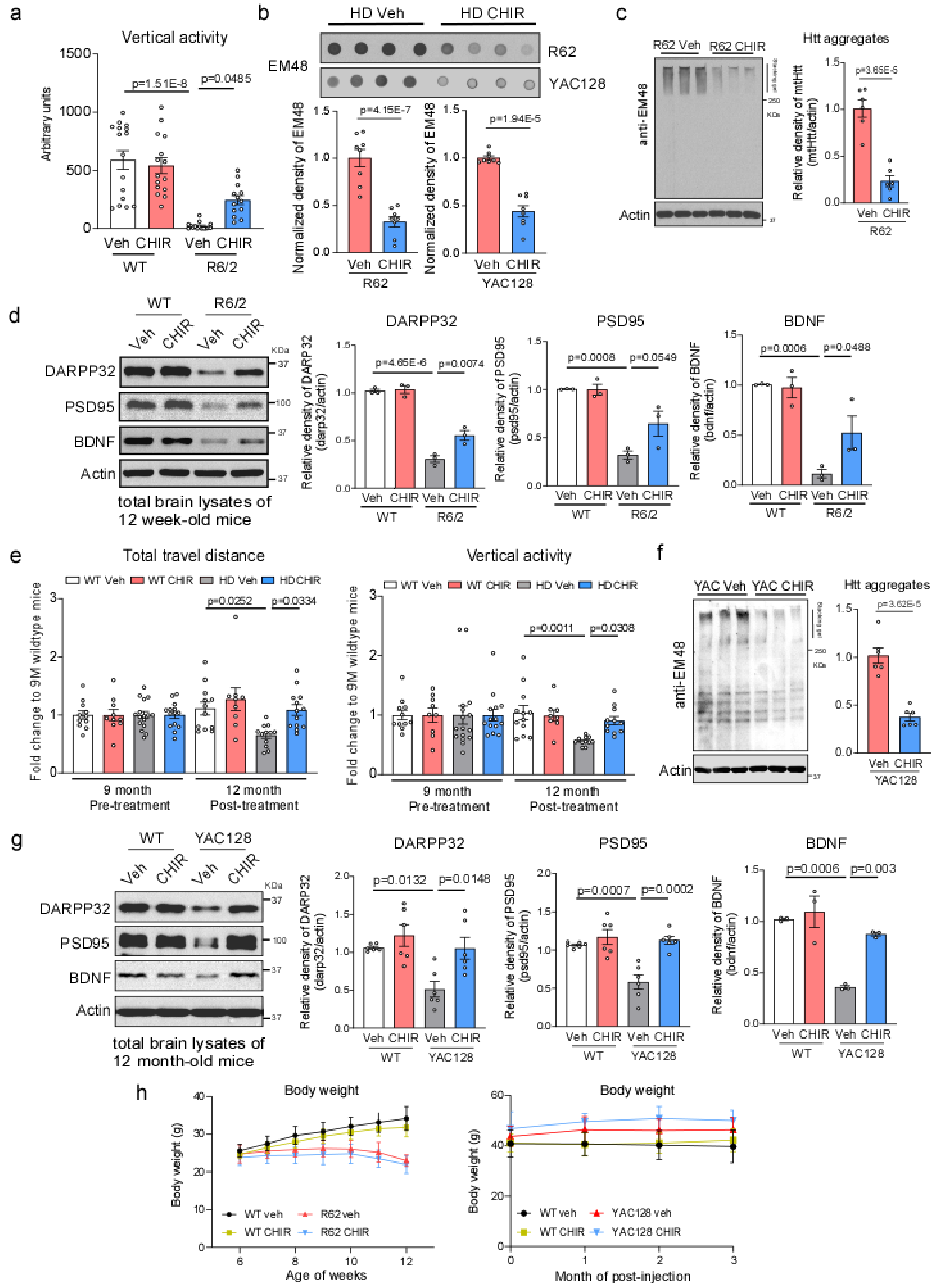

Supplementary Figure. 2. CHIR99021 is protective in HD mice. CHIR99021 was given by intraperitoneal (i.p) injection from 6- to 12-weeks in R6/2 mice. (**a**) Mouse movement activity (1 h) was assessed using the open-field activity chamber at 9-weeks old. The vertical activity of mice is shown. WT Veh n = 16, WT CHIR n = 15, R6/2 Veh n = 15, R6/2 CHIR n = 13 mice/group. (**b**) Striatum was harvested from 12-week-old R6/2 and 12-month-

old YAC128 mice, and mtHtt aggregates in triton-insoluble fractions were examined using an anti-Htt antibody (clone EM48) by dot blot assay. Histogram: the relative abundance of mtHtt protein aggregates. n = 8 mice/group. (**c**) Triton-insoluble fractions from the striatum of 12-week-old R6/2 mice were subjected to western blotting (WB). n = 6 mice/group. (**d**) Total lysates from the striatum of 12-week-old R6/2 mice and WT littermates were subjected to WB. Histogram: the relative abundance of DARPP-32, PSD95 and BDNF. CHIR99021 (i.p injection) was given from 9–12 months old YAC128 mice. n = 3 mice/group (**e**) Mouse movement activity (24 h) was determined. WT Veh n = 10, WT CHIR n = 10, HD Veh:n = 16 (9 month) or 12 (12 month), HD CHIR n = 14 mice/group. (**f**) Striatal triton-insoluble fractions of 12-month-old YAC128 mice were subjected to WB. n = 6 mice/group. (**g**) Total lysates from the striatum of 12-month-old YAC128 mice and WT littermates were subjected to WB. Histogram: the relative abundance of DARPP-32 (n = 6), PSD95 (n = 6), and BDNF (n = 3). (**h**) Left: body weight of R6/2 and WT littermates was recorded at 6–12 weeks old. n = 10 mice/group. Right: body weight of YAC128 and WT littermates was recorded at 0–3 month-old post-injection. WT Veh n = 10, WT CHIR n = 10, YAC128 Veh n = 11, YAC128 CHIR n = 13 mice/group. Data were the mean ± SEM and compared using one-way ANOVA with Tukey's *post-hoc* test in a, d, e, g, and h, and unpaired student *t*-test (two-tailed) in b, c, and f. Exact p values are shown in the figures.

Supplementary Fig. 3

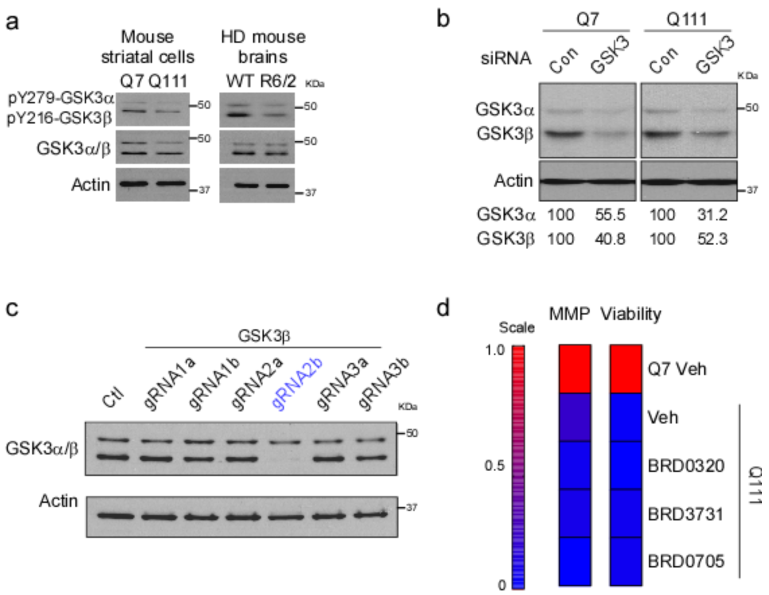

Supplementary Figure. 3. Cellular targets other than GSK3 mediate CHIR99021 protection in HD. (**a**) Total protein lysates were harvested from HdhQ7 and HdhQ111 striatal cells, and striatum from 12-week-old R6/2 and WT mice. Western blotting was performed with indicated antibodies. (**b**) Representative immunoblots for *GSK3α/β* in HdhQ7 and HdhQ111 cells expressing control (Con) or GSK3 small interfering RNA (siRNA). (**c**) *GSK3β* knockout (KO) using CRISPR-Cas9 in Neuro2a cells using multiple guide RNAs (gRNAs). Representative immunoblots showing KO efficiency in Neuro2a clones using different gRNAs; gRNA2b for GSK3 was used in this study. HdhQ111 striatal cells were treated with highly specific GSK3 inhibitors (BRD0320, BRD3731, and BRD0705) for 2 days. (**d**) MMP and cell viability results were normalized to HdhQ7 cells and represented in a dual-color heat map. Scale: 0–1. MMP was measured by TMRM staining; cell death was measured by LDH release after 16 h serum starvation. Data are representative of two independent experiments for a, b, c.

Supplementary Fig. 4

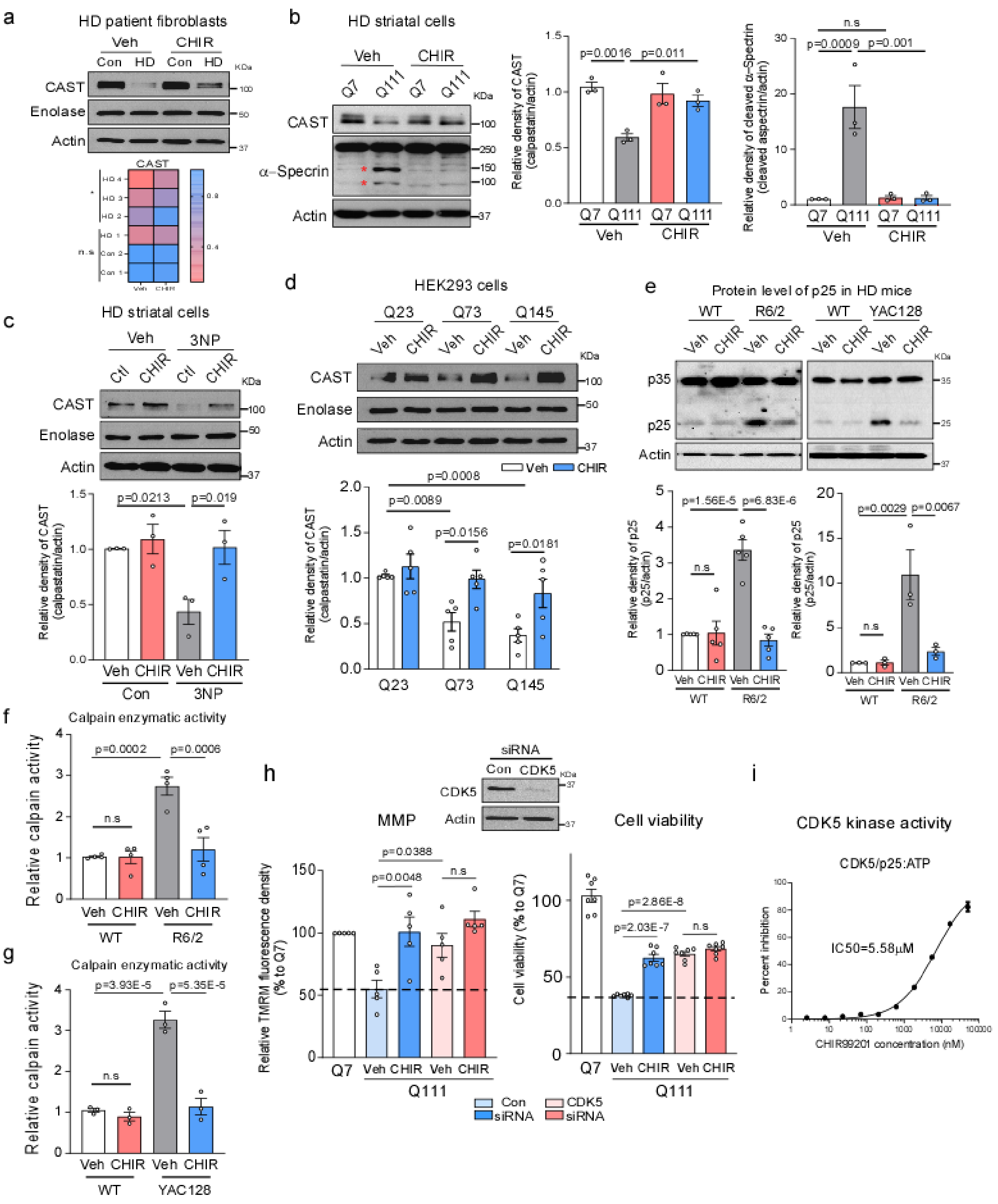

Supplementary Figure. 4. CHIR99201 mitochondrial protection is dependent on the CAST-calpain pathway. (**a**) Total lysates of fibroblasts from control subjects (Con; HDF, Hufl) or patients with HD (4693, 21756, 3621, 5539) were subjected to western blotting (WB). Heat map: CAST protein relative abundance in control and HD fibroblasts. Protein abundance range: 0–1. * $p < 0.05$ vehicle (Veh)-treated fibroblasts versus CHIR99021-treated fibroblasts (p value HD2: 0.016, HD3: 0.019, HD4: 0.031). (**b**) Total lysates of HdhQ7 and HdhQ111 striatal cells treated with Veh or CHIR99021 were subjected to WB. * indicates cleaved α-spectrin. Histogram: CAST and cleaved α-spectrin protein relative abundance. (**c**) HdhQ7 cells were treated with 3-nitropropionic acid (3-NP, 5 mM) followed by CHIR99021 (3 µM) treatment. Total lysates were harvested from cells 48 h after 3-NP treatment and subjected to WB. Histogram: CAST relative protein abundance. (**d**) Total

lysates were harvested from HEK293T cells expressing Myc-Q23-Htt, Myc-Q73-Htt or Myc-Q145-Htt after treatment with Veh or CHIR99021. WB was performed. Top: representative immunoblots. Bottom: CAST relative abundance. (**e**) Total lysates were harvested from the striatum of HD mice (left: R6/2 at 12 weeks old, n = 5, right: YAC128 at 12 months old, n = 3) treated with Veh or CHIR99021. WB was performed. Histogram: p25 relative protein abundance. Calpain relative enzymatic activity was measured in (**f**) R6/2 and WT littermates (n = 4), and (**g**) YAC128 and WT littermates (n = 3). (**h**) HdhQ111 cells were transfected with control (Con) siRNA or cyclin-dependent kinase 5 (CDK5) siRNA for 3 days. CDK5 knockdown efficiency was determined by WB. Left: MMP was measured by TMRM staining (n = 5). Right: cell viability was measured by MTT assay (n = 7). (**i**) In vitro CDK5 activity was assessed in the presence of p25 protein. The IC_{50} of CHIR99021 toward CDK5 activity was calculated. Data are reported as the mean ± SEM and analyzed using one-way ANOVA with Tukey's *post-hoc* test in a–c, e–h, and two-way ANOVA with Šídák's *post-hoc test* in d. Exact p values are shown in the figures. Data are representative of at least three independent experiments.

Supplementary Fig. 5

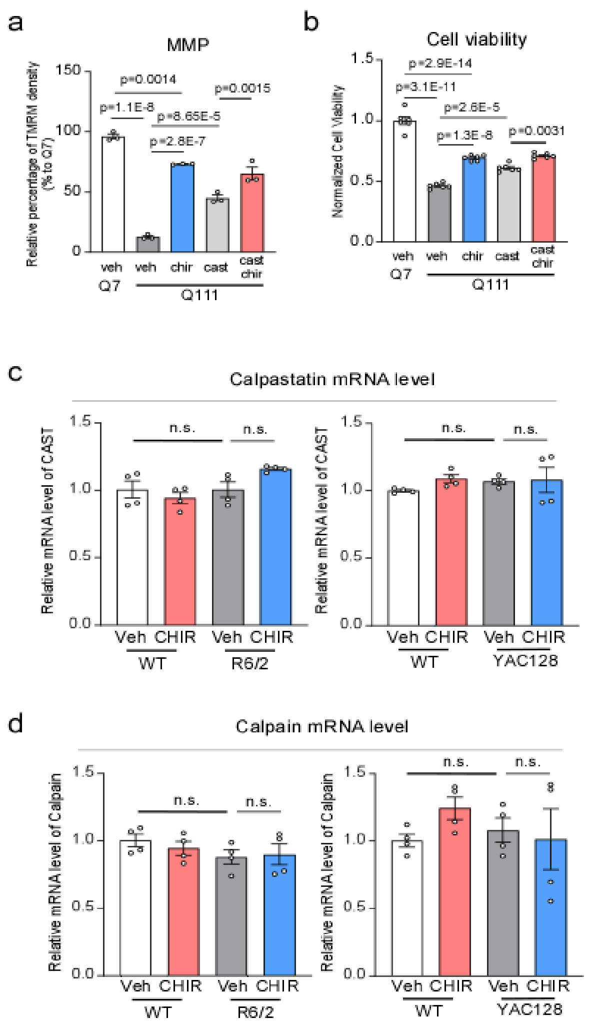

Supplementary Figure. 5. CHIR99021 treatment affects the CAST-calpain pathway in HD. HdhQ7 and HdhQ111 cells were treated with 3 µM CHIR99021 and/or 5 µM N-acetyl-calpastatin peptide for 48 h. (**a**) MMP was assessed by TMRM staining (n = 3 biologically independent samples). (**b**) Cell viability was measured by MTT assay after 16 h serum starvation (n = 6 biologically independent samples). The mRNA levels of (**c**) calpastatin and (**d**) calpain in vehicle (Veh)- or CHIR99021-treated WT and HD mice were examined by qPCR (n = 4 mice/group). All values are reported as the mean ± SEM from at least three independent experiments and analyzed using one-way ANOVA with Tukey's *post-hoc* test. Exact p values are shown in the figures.

Supplementary Fig. 6

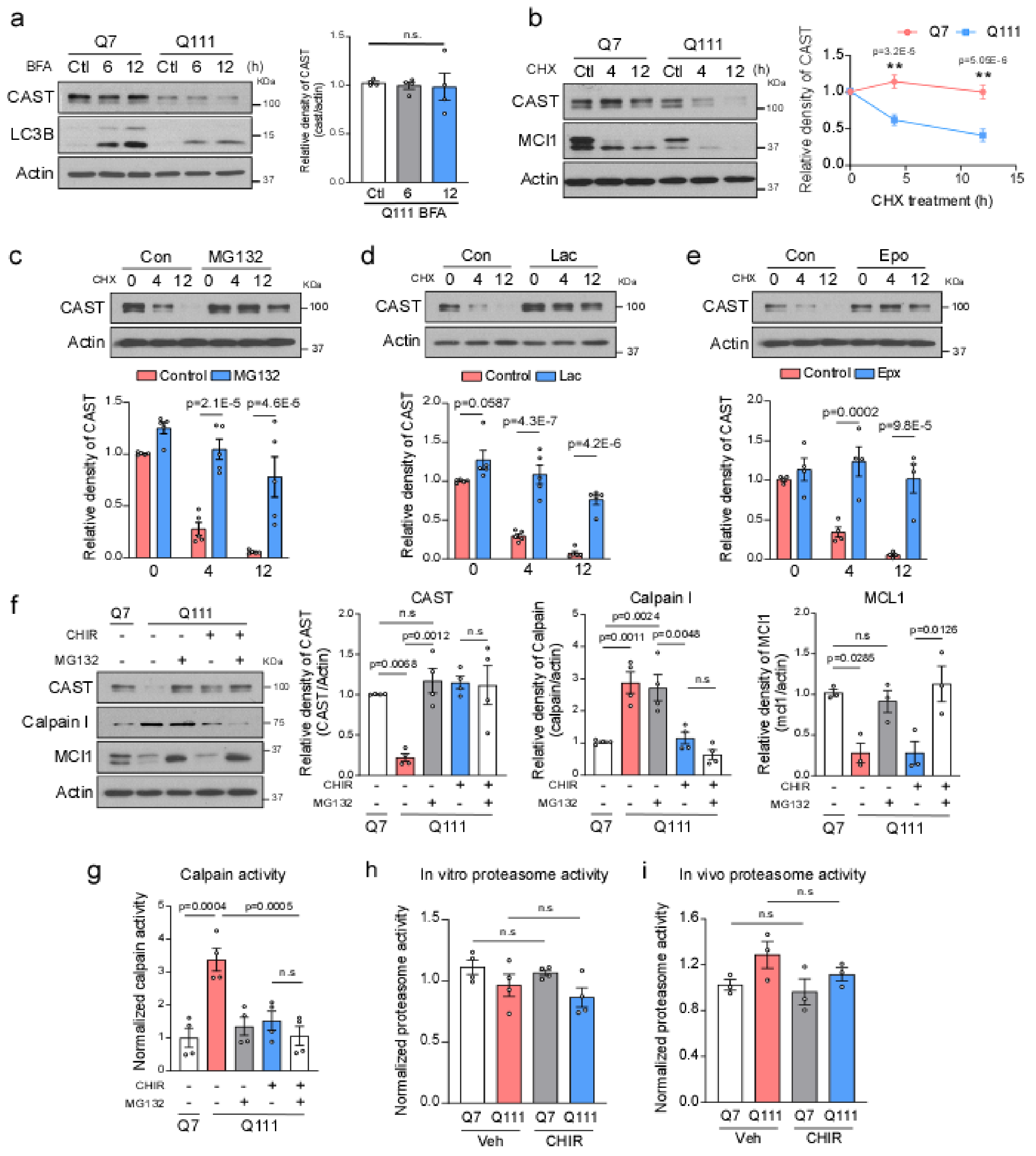

Supplementary Figure. 6. Ubiquitin-proteasome system (UPS)-mediated degradation of CAST in HD. (**a**) Total lysates were harvested from HdhQ7 and HdhQ111 cells after 6 or 12 h treatment with 20 µM bafilomycin A1 (BFA) and subjected to western blotting (WB). Left: representative immunoblots. Right: CAST protein relative abundance (n = 4). (**b**) Total lysates were harvested from HdhQ7 and HdhQ111 striatal cells treated with 100 µg/mL cycloheximide (CHX), and subjected to WB. Left panel: representative immunoblots. Right panel: CAST protein relative abundance after CHX treatment. Total lysates were harvested from HdhQ111 cells treated with CHX together with (**c**) MG132 (n = 5) or (**d**) lactacystin (Lac) (n = 5) or (**e**) epoxomicin (Epo) (n = 4) and subjected to WB with indicated antibodies. Histogram: CAST relative protein abundance. (**f**) Total lysates of HdhQ7 and HdhQ111 cells were harvested after indicated treatments: 3 µM CHIR99021 for 48 h followed by MG132 treatment for 12 h and subjected to WB with indicated antibodies. Histograms: CAST (n = 4), calpain I (n = 4) and MCL1 (n = 3) relative protein abundance. (**g**) Calpain enzymatic activity was examined in HdhQ7 and

HdhQ111 cells at indicated treatments: 3 μM CHIR99021 for 48 h followed by MG132 for 12 h (n = 4). (**h**) The total proteasome fraction was extracted from HdhQ7 and HdhQ111 cells and incubated with vehicle (Veh) or CHIR99021 for 30 min followed by enzymatic activity assessment (n = 4). (**i**) The total proteasome fraction was extracted from HdhQ7 and HdhQ111 cells treated with Veh or CHIR99021 for 48 h and subjected to proteasome activity assay (n = 3). All values are reported as the mean ± SEM and analyzed using one-way ANOVA with Tukey's *post-hoc* test in a, f–i, and two-way ANOVA with Šídák's *post-hoc test in* b–e. Data are representative of at least three independent experiments. Exact p values are shown in the figures.

Supplementary Fig. 7

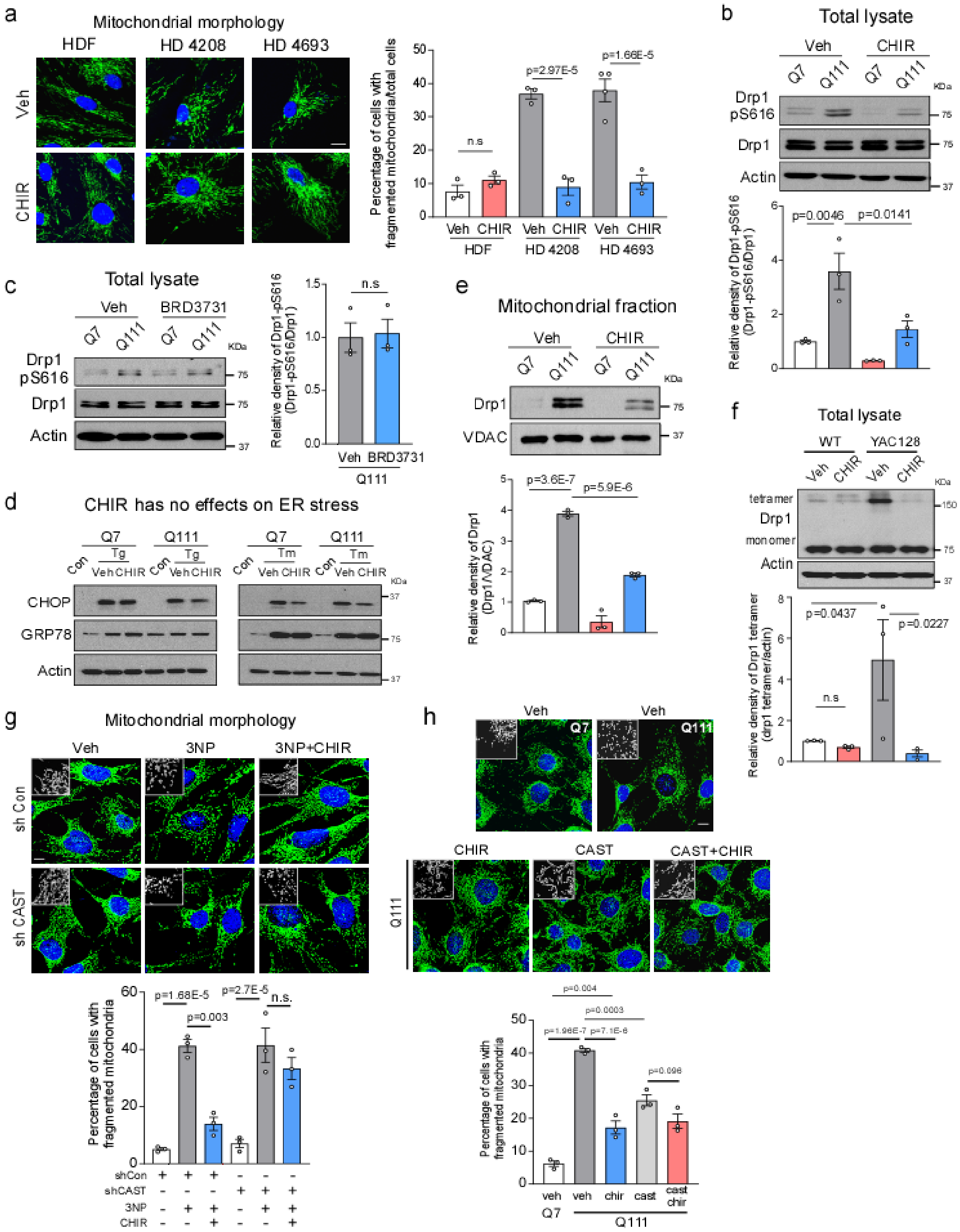

Supplementary Figure. 7. Drp1-mediated mitochondrial fragmentation is a key downstream pathway of CHIR99021 in HD. (**a**) Representative anti-Tom20 staining images in control fibroblast (HDF) and HD patient fibroblasts (4208, 4693) treated with vehicle (Veh) or CHIR99021 at 3 μM. Scale bar = 30 μm. Histogram: the percentage of cells with fragmented mitochondria. At least 100 cells/group were counted. (**b**) Cells were treated with Veh or CHIR99021 for 48 h, and total lysates of cells were subjected to western blotting (WB). Histogram: the relative protein abundance of phosphor-Ser616 Drp1 (Drp1-pS616). (**c**) Total lysates were harvested from HdhQ7 and HdhQ111 cells treated with Veh or BRD3731 (3 μM) for 48 h and subjected to WB. Histogram: the relative density of Drp1-pS616. (**d**) Representative immunoblots of CHOP and GRP78 in HdhQ7 and HdhQ111 striatal cells after indicated treatments: 3 μM CHIR99021 for 48 h, 5 μM thapsigargin (Tg) for 8 h, and 10 μg/mL tunicamycin (Tm) for 16 h. (**e**) Mitochondrial fractions were harvested from HdhQ7 and HdhQ111 cells treated with Veh or CHIR99021 for 48 h and subjected to WB. Histogram: the relative protein abundance of Drp1. (**f**) Total lysates were harvested from the striatum of 12-month-old YAC128 and WT mice treated with Veh or CHIR99021. Drp1 tetramer levels were examined by WB in the absence of β-mercaptoethanol. Histogram: the relative abundance of the Drp1 tetramer. (**g**) Representative anti-Tom20 staining images in control shRNA (Con sh)- or CAST shRNA (CAST sh)-expressing HdhQ7 cells treated with 3-nitropropionic acid (3-NP) and/or CHIR99021. Histogram: the percentage of cells with fragmented mitochondria. At least 100 cells/group were counted. Scale bar = 30 μm. (**h**) Representative anti-Tom20 staining images in HdhQ7 and HdhQ111 cells with indicated treatments: N-acetyl-calpastatin (CAST) for 48 h and CHIR99021 for 48 h. Scale bar = 30 μm. Histogram: the percentage of cells with fragmented mitochondria. At least 100 cells/group were counted. Data are expressed as the mean ± SEM and analyzed using one-way ANOVA with Tukey's *post-hoc* test. Data are representative of at least three independent experiments. Exact p values are shown in the figures.

Supplementary Table 1. Primer sequences used for qPCR amplification

**Primer**	**Sequence**	**PCR conditions**
m*GAPDH*	s: GACTTCAACAGCAACTCCCAC<br>as: TCCACCACCCTGTTGCTGTA	50°C, 2 min<br>95°C, 2 min<br>followed by 40 cycles of<br>95°C, 15 s<br>60°C, 1 min
m*CAPN1*	s: ATGACAGAGGAGTTAATCACCCC<br>as: GGCTATGAGAAACCGGAGGG	
m*CAST*	s: GGAAGGACAAACCAGAGAAGC<br>as: AGGGGCAGCTATCCAAATCTT	
