# Supplementary material for: Small-molecule suppression of calpastatin degradation reduces neuropathology in models of Huntington’s disease
Source: Nat Commun. 2021 Sep 6;12:5305. doi: 10.1038/s41467-021-25651-y (PMC8421361; doi:10.1038/s41467-021-25651-y)
Supplement: Supplementary file 3 — Reporting Summary [file 41467_2021_25651_MOESM3_ESM.pdf]

nature portfolio

Corresponding author(s): Xin Qi

Last updated by author(s): Aug 5, 2021

Reporting Summary

Nature Portfolio wishes to improve the reproducibility of the work that we publish. This form provides structure for consistency and transparency in reporting. For further information on Nature Portfolio policies, see our Editorial Policies and the Editorial Policy Checklist.

Statistics

For all statistical analyses, confirm that the following items are present in the figure legend, table legend, main text, or Methods section.

n/a	Confirmed	
☐	☒	The exact sample size (n) for each experimental group/condition, given as a discrete number and unit of measurement
☐	☒	A statement on whether measurements were taken from distinct samples or whether the same sample was measured repeatedly
☐	☒	The statistical test(s) used AND whether they are one- or two-sided<br>*Only common tests should be described solely by name; describe more complex techniques in the Methods section.*
☐	☒	A description of all covariates tested
☐	☒	A description of any assumptions or corrections, such as tests of normality and adjustment for multiple comparisons
☐	☒	A full description of the statistical parameters including central tendency (e.g. means) or other basic estimates (e.g. regression coefficient) AND variation (e.g. standard deviation) or associated estimates of uncertainty (e.g. confidence intervals)
☒	☐	For null hypothesis testing, the test statistic (e.g. F, t, r) with confidence intervals, effect sizes, degrees of freedom and P value noted<br>*Give P values as exact values whenever suitable.*
☒	☐	For Bayesian analysis, information on the choice of priors and Markov chain Monte Carlo settings
☒	☐	For hierarchical and complex designs, identification of the appropriate level for tests and full reporting of outcomes
☒	☐	Estimates of effect sizes (e.g. Cohen's d, Pearson's r), indicating how they were calculated

Our web collection on statistics for biologists contains articles on many of the points above.

Software and code

Policy information about availability of computer code

Data collection	FV10-ASW 4.2 Viewer (for confocal imaging), Keyence BZ-X viewer (for Keyence BZ-X700)
Data analysis	GraphPad Prism 9.0 (for quantification and statistic analysis), Scaffold 4.4.0 (for proteomics analysis), Fiji ImageJ 1.5 (for image analysis)

For manuscripts utilizing custom algorithms or software that are central to the research but not yet described in published literature, software must be made available to editors and reviewers. We strongly encourage code deposition in a community repository (e.g. GitHub). See the Nature Portfolio guidelines for submitting code & software for further information.

Data

Policy information about availability of data

All manuscripts must include a data availability statement. This statement should provide the following information, where applicable:

- Accession codes, unique identifiers, or web links for publicly available datasets
- A description of any restrictions on data availability
- For clinical datasets or third party data, please ensure that the statement adheres to our policy

Data supporting the findings of this study are provided within the paper and its supplementary information. Source data are provided with this paper and all statistical data are presented in Source Data file. The proteomic database was submitted to figshare (https://figshare.com/) with DOI: 10.6084/m9.figshare.14850267.v1. Hyperlink: https://doi.org/10.6084/m9.figshare.14850267.v1

Field-specific reporting

Please select the one below that is the best fit for your research. If you are not sure, read the appropriate sections before making your selection.

- [x] Life sciences
- [ ] Behavioural & social sciences
- [ ] Ecological, evolutionary & environmental sciences

For a reference copy of the document with all sections, see nature.com/documents/nr-reporting-summary-flat.pdf

Life sciences study design

All studies must disclose on these points even when the disclosure is negative.

Sample size	The sample size per group was determined from previous publications with similar methodologies (Zhao et al., 2019, Nature communications; Guo et al., 2016, Nature communications; Guo et al., 2013, J. Clin. Invest).
Data exclusions	No data were excluded from the analysis
Replication	Replication of experiments was successful in three different experiments/cohort and data is accumulated during the analysis process. Results also were replicated in independent experimental setting.
Randomization	HD mice and their littermate WT mice were collected from each cohort depending on the genotype and used for the experiment. All WT and HD animals were assigned randomly to vehicle or CHIR99021 treatment, and within animal controls were performed wherever possible. All cells analyzed for quantification were randomly selected. Strains of iPS-cells from normal subject and HD patients were randomly selected with different CAG repeats, for neuronal differentiation and further experiments. All mouse samples were randomly selected for experiments including: IHC, qPCR, western blot, calpain activity assay, proteasome activity assay. Frozen tissue from normal subject and HD patients were randomly diced and homogenated for experiments.
Blinding	The investigators were blinded to group allocation during data collection and analysis.

Reporting for specific materials, systems and methods

We require information from authors about some types of materials, experimental systems and methods used in many studies. Here, indicate whether each material, system or method listed is relevant to your study. If you are not sure if a list item applies to your research, read the appropriate section before selecting a response.

Materials & experimental systems

n/a	Involved in the study	
☐	☒	Antibodies
☐	☒	Eukaryotic cell lines
☒	☐	Palaeontology and archaeology
☐	☒	Animals and other organisms
☒	☐	Human research participants
☒	☐	Clinical data
☒	☐	Dual use research of concern

Methods

n/a	Involved in the study	
☒	☐	ChIP-seq
☒	☐	Flow cytometry
☒	☐	MRI-based neuroimaging

Antibodies

Antibodies used	anti-DARPP-32 (ab40801, Abcam, Cambridge, UK, 1:3000), anti-BDNF (Abcam, 1:1000), anti-PGC1α (ab54481, Abcam, 1:1000), anti-VDAC1 (14734, Abcam, 1:2000), anti-CDK5 (ab40773, Abcam, 1:1000), anti-MCL1 (16225-1-AP, Proteintech, Rosemont, IL, USA, 1:1000), anti-Mff (17090-1-AP, Proteintech, 1:2000), anti-p62 (18420-1-AP, Proteintech, 1:5000), anti-Fis1 (10956-1-AP, Proteintech, 1:1000), anti-MIEF (20164-1-AP, Proteintech, 1:1000), anti-TFEB (13372-1-AP), anti-ATPB (17247-1-AP, Proteintech, 1:3000), anti-MFN1 (H00055669-M04, Abnova, Taipei, Taiwan, 1:2000), anti-GRP78 (SPA-827, Stressgen Enzo Life Sciences, Farmingdale, NY, USA, 1:1000), anti-GSK3 (05-412, MilliporeSigma, 1:1000), anti-huntingtin protein (MAB5374, clone EM48, MilliporeSigma, 1:1000), anti-β-actin (A1978, MilliporeSigma, 1:10000), anti-DLP1 (611113, BD Bioscience, Franklin Lakes, NJ, USA, 1:2000), anti-GSK3 pY216/279 (612312, BD Bioscience, 1:1000), anti-SOD2 (611580, BD Bioscience, 1:1000), anti-OPA1 (612607, BD Bioscience, 1:2000) anti-PSD95 (2507, Cell Signaling, Danvers, MA, USA, 1:5000), anti-Drp1-pS616 (3455S, Cell Signaling, 1:1000), anti-p35/25 (2680, Cell Signaling, 1:1000), anti-LC3 (2775S, Cell Signaling, 1:1000), anti-CAST (4146, Cell Signaling, 1:2000), anti-calpain (2556, Cell Signaling, 1:1000), anti-ubiquitin (43124S, Cell Signaling, 1:1000), anti-spectrin-α II (sc-48382, Santa Cruz, Dallas, TX, USA, 1:500), anti-CHOP (sc575, Santa Cruz, 1:2000), and anti-enolase (sc-15343, Santa Cruz, 1:2000), anti-DARPP-32 (1710-1, Epitomics, Burlingame, CA, USA; 1:500), anti-Tau (T1308-1, rPeptide, Athens, Georgia, USA 1:200), anti-DARPP-32 (ab40801, Abcam, 1:500), anti-MAP2 (4542, Cell Signaling, 1:500), anti-GAD67 (MAB5406, MilliporeSigma, 1:300), anti-Tubulin β 3 (Tuj1) (801201, BioLegend, San Diego, CA, USA, 1:500), and anti-Tom20 (11802-1-AP, Proteintech, 1:1000).HRP-conjugated anti-rabbit or anti-mouse IgG (31430/31460, ThermoFisher Scientific, 1:5000). Alexa 488, goat anti-mouse Ig G (H+L) (A11029, Invitrogen, 1;1000), Alexa 488, goat anti-rabbit Ig G (A11034, Invitrogen,

	1:1000), Alexa 568, goat anti-mouse Ig G (H+L) (A11031, Invitrogen, 1:1000), Alexa 568, goat anti-rabbit, Ig G (H+L) (A11036, Invitrogen, 1:1000), Alexa Fluor® 405 Goat Anti-Mouse IgG (H+L) (A31553, Invitrogen, 1:1000).
Validation	anti-DARPP-32 (PMID: 33933677), anti-BDNF (PMID: 31668016), anti-PGC1α (PMID: 33146548), anti-VDAC1 (PMID: 33164581), anti-CDK5 (PMID: 32353462), anti-MCL1 (PMID: 30678274), anti-Mff (PMID: 30581454), anti-p62 (PMID: 30654731), anti-Fis1 (PMID: 30587587), anti-MIEF (PMID: 30587587), anti-TFEB (PMID: 31261758), anti-ATPB (PMID: 30759120), anti-MFN1 (PMID: 32898935), anti-GRP78 (PMID: 33661766), anti-GSK3 (PMID: 25764078), anti-huntingtin protein (PMID: 25859666), anti-β-actin (PMID: 24687991), anti-DLP1 (PMID: 17003040), anti-GSK3 pY216/279 (PMID: 10995469), anti-SOD2 (PMID: 9973207), anti-OPA1 (PMID: 11017080), anti-PSD95 (PMID: 33483466), anti-Drp1-pS616 (PMID: 33479191), anti-p35/25 (PMID: 33285637), anti-LC3 (PMID: 33664298), anti-CAST (PMID: 33323513), anti-calpain (PMID: 33323513), anti-ubiquitin (PMID: 32424132), anti-spectrin-α II (PMID: 34080307), anti-CHOP (PMID: 26963025), anti-enolase (PMID: 26268247), anti-MAP2 (PMID: 33302995), anti-GAD67 (PMID: 26441578), anti-Tubulin β 3 (PMID: 28281529), anti-Tom20 (PMID: 30598479), anti-Tau (PMID: 18987184)

Eukaryotic cell lines

Policy information about cell lines

Cell line source(s)	HEK293T from ATCC (CRL-1573), Neuro2A from ATCC (CCL-131), HdhQ7/HdhQ111 from CHDI, human fibroblasts from Coriell institute, iPSC from NIH human cell and data repository (NHCDR)
Authentication	all cell lines were authenticated by SNP test.
Mycoplasma contamination	cell lines were tested negative for mycoplasma contamination.
Commonly misidentified lines (See ICLAC register)	No commonly misidentified cell lines were used.

Animals and other organisms

Policy information about studies involving animals; ARRIVE guidelines recommended for reporting animal research

Laboratory animals	4 weeks-old Male R6/2 (B6CBA-Tg(HDexon1)62Gpb/3J) (Stock No: 006494) and WT littermates were purchased from Jackson Laboratory. IP injection of CHIR99021 were given at age of 6-12 weeks-old.<br>YAC128 (FVB-Tg (YAC128) 53Hay/J, JAX stock number: 004938) breeders (FVB/N genetic background) were also purchased from The Jackson Laboratory. 2-4 months-old mice were used for breeding and 1 month-old mice were used for genotyping. IP injection of CHIR99021 were given for male YAC128 mice at age of 9-12 months-old.<br>Mice were maintained in rooms with 12 light/12 dark cycle, 23°C, 40-60% humidity, and food/water accessible at all times
Wild animals	study did not involve wild animals
Field-collected samples	study did not involve the sample collected in fields
Ethics oversight	All animal studies were conducted in accordance with protocols approved by the Institutional Animal Care and Use Committee of Case Western Reserve University, and were performed based on the NIH Guide for the Care and Use of Laboratory Animals

Note that full information on the approval of the study protocol must also be provided in the manuscript.
